# Hypertension Susceptibility Loci are Associated with Anthracycline-related Cardiotoxicity in Long-term Childhood Cancer Survivors

**DOI:** 10.1038/s41598-017-09517-2

**Published:** 2017-08-29

**Authors:** Michelle A. T. Hildebrandt, Monica Reyes, Xifeng Wu, Xia Pu, Kara A. Thompson, Jianzhong Ma, Andrew P. Landstrom, Alanna C. Morrison, Joann L. Ater

**Affiliations:** 1 Department of Epidemiology, University of Texas MD Anderson Cancer Center, Houston, Texas, USA; 2 Department of Cardiology, University of Texas MD Anderson Cancer Center, Houston, Texas, USA; 3 Division of Pediatrics, University of Texas MD Anderson Cancer Center, Houston, Texas, USA; 4 Department of Epidemiology, Human Genetics and Environmental Sciences, University of Texas School of Public Health, Houston, Texas, USA; 5 Section of Cardiology, Department of Pediatrics, Baylor College of Medicine, Houston, Texas, USA

## Abstract

Anthracycline-based chemotherapy is associated with dose-dependent, irreversible damage to the heart. Childhood cancer survivors with hypertension after anthracycline exposure are at increased risk of cardiotoxicity, leading to the hypothesis that genetic susceptibility loci for hypertension may serve as predictors for development of late cardiotoxicity. Therefore, we determined the association between 12 GWAS-identified hypertension-susceptibility loci and cardiotoxicity in a cohort of long-term childhood cancer survivors (N = 108) who received anthracyclines and were screened for cardiac function via echocardiograms. Hypertension-susceptibility alleles of *PLCE1*:rs9327264 and *ATP2B1*:rs17249754 were significantly associated with cardiotoxicity risk conferring a protective effect with a 64% (95% CI: 0.18–0.76, P = 0.0068) and 74% (95% CI: 0.07–0.96, P = 0.040) reduction in risk, respectively. In RNAseq experiments of human induced pluripotent stem cell (iPSC) derived cardiomyocytes treated with doxorubicin, both *PLCE1* and *ATP2B1* displayed anthracycline-dependent gene expression profiles. *In silico* functional assessment further supported this relationship - rs9327264 in *PLCE1* (P = 0.0080) and *ATP2B1* expression (P = 0.0079) were both significantly associated with daunorubicin IC_50_ values in a panel of lymphoblastoid cell lines. Our findings demonstrate that the hypertension-susceptibility variants in *PLCE1* and *ATP2B1* confer a protective effect on risk of developing anthracycline-related cardiotoxicity, and functional analyses suggest that these genes are influenced by exposure to anthracyclines.

## Introduction

There has been tremendous success over the past 40 years in the treatment of childhood cancers that has resulted in a dramatic shift in the 5-year survival rate for these patients, from less than 60% in the 1970s to over 80% in more recent reports^1^. This improvement can be attributed, in part, to advancements in treatments; this includes anthracyclines that are used in the treatment of over 50% of childhood cancer patients. Unfortunately, the use of these agents leads to dose-dependent progressive and permanent damage to the heart in up to half of patients^2, 3^. However, it is clear that a number of survivors develop clinical cardiovascular side effects at current recommended dosing, with a much higher proportion exhibiting subclinical cardiotoxicity^3, 4^. This underscores the need for predictive biomarkers to enable identification of those at high risk who would be candidates for alternative therapeutic regimens and/or cardioprotective interventions, as well as to guide the design of risk-stratified, cost-effective follow-up surveillance programs to reduce the adverse effects of anthracyclines on the heart.

It has been established that cytotoxicity to anthracyclines is a heritable trait, with 20–60% of the variation being accounted for by genetic factors depending on the dose^5^. Genome-wide association studies have implicated variants in *RARG* and *CELF4* variant as potential mediators of anthracycline-related cardiotoxicity^6, 7^. Several other studies have focused on the identification of genetic predictors using a candidate gene-based approach^8–12^. However, the full spectrum of genetic mediators of anthracycline-related cardiotoxicity in childhood cancer survivors remain undiscovered.

In a study of modifiable risk factors for late cardiotoxicity, hypertension had the highest relative excess risk due to interactions with anthracyclines when compared to other risk factors including dyslipidemia, diabetes, and obesity^13^. In order to further explore the relationship between hypertension and anthracycline exposure, we hypothesized that genetic variants related to hypertension would help predict anthracycline-related cardiotoxicity. Hypertension is typically not a disease of adolescents and young adults, regardless of their genetic predisposition. However, the combination of exposure to anthracyclines and hypertension genetic susceptibility loci puts this subgroup of individuals at greater risk of developing cardiotoxicity. A large scale meta-analysis of blood pressure in over 200,000 individuals identified 12 genetic loci highly significant for hypertension^14^. To identify predictors of anthracycline-induced cardiotoxicity, we genotyped a cohort of long-term childhood cancer survivors for these loci. They had all been treated with anthracyclines and were followed for a median of 15.8 years with echocardiogram-based screening according to COG guidelines. The genetic association findings were then followed up with analysis of gene expression in iPSC-cardiomyocytes exposed to anthracyclines and *in silico* functional assessment.

## Results

### Patient Population

A total of 108 long-term childhood cancer survivors were included in this analysis, of which 46 were classified as having cardiotoxicity during the median follow-up time of 15.82 years (Table 1). The populations were well matched by age at diagnosis, gender, anthracycline dose, chest radiation, and cancer type. Cases had a significantly decreased ejection fraction (EF) compared to cases (43.48 vs, 57.14, P < 0.0001). There was a difference in the cases and controls by race with slightly more Hispanic survivors with an event (P = 0.028) and the follow-up time was slightly longer in the cases than controls (21.20 vs. 15.66 years). More of the cases were diagnosed with hypertension during follow-up compared to controls (59% vs. 35%, P = 0.017) and a diagnosis of hypertension was associated with a 2.58-fold increased risk of cardiotoxicity (95% CI: 1.18–5.66, P = 0.018). Overall, average heart failure risk score was 5.7, placing our population in the “high-risk” group (Table 1).

**Table 1:** Host Characteristics.

Variable	Cases n(%)	Controls n(%)	P-value
**Total**	**46**	**62**	
Age at diagnosis, mean(SD)	9.2(4.7)	9.3(5.7)	0.92
Gender			
Female	21(46)	32(52)	0.54
Male	25(54)	30(48)	
Race			
White	22(48)	38(61)	0.028
Hispanic	16(35)	10(16)	
Black	8(17)	9(15)	
Other	0(0)	5(8)	
Chest radiation			
No	31(67)	48(77)	0.24
Yes	15(33)	14(23)	
Cancer site			
Sarcoma	21(46)	20(32)	0.092
Leukemia	5(11)	19(31)	
Lymphoma	14(30)	15(24)	
Other	6(13)	8(13)	
Anthracycline cumulative dose, mean(SD)	319.5(111.5)	273.9(157.6)	0.10
Follow-up time in years, mean(SD)	21.2(11.2)	15.7(7.6)	0.0027
Average EF% low*, mean(SEM)	43.48(1.152)	57.14(0.28)	< 0.0001
Risk score, mean(SD)	6.0(1.7)	5.4(1.4)	0.032
Hypertension			
No	19(41)	40(65)	0.017
Yes	27(59)	22(35)	

*Average of two lowest EF% for each patient.

### Cardiotoxicity Risk

Of the 12 variants previously identified to be associated with increased risk of hypertension, two were also statistically significant for risk of cardiotoxicity. *PLCE1*:rs932764 was significantly associated with cardiotoxicity risk in both univariate and multivariate analyses (Table 2). Under the additive model, the hypertension risk allele conferred a 64% reduction in cardiotoxicity risk (95% CI: 0.18–0.76, P = 0.0068). Similarly, the association between *ATP2B1*:rs17249754 and cardiotoxicity risk was protective with carriers of at least one of the hypertension risk alleles having an approximately 74% reduction in risk of cardiotoxicity (95% CI: 0.07–96, P = 0.040).

**Table 2:** Hypertension-susceptibility Variants and Associations with Cardiotoxicity in Long-term Childhood Cancer Survivors.

Gene	Chr.	Variant	Variant Location	Hypertension Risk Allele	Hypertension Risk AF	Model	OR(95% CI)	P-value	*OR(95% CI)	P-value
*PLCE1*	10	rs932764	intron	G	0.45	add	0.48(0.27–0.85)	**0.012**	0.36(0.18–0.76)	**0.0068**
*ATP2B1*	12	rs17249754	5’ flanking	G	0.90	rec	0.33(0.12–0.92)	**0.034**	0.26(0.07–0.96)	**0.040**
*ARHGAP42*	11	rs633185	intron	C	0.64	add	1.32(0.72–2.43)	0.37	1.93(0.84–4.43)	0.12
*GNAS-EDN3*	20	rs6015450	intergenic	G	0.13	dom	1.64(0.67–4.03)	0.28	2.21(0.72–6.80)	0.17
*C10orf107*	10	rs4590817	intron	G	0.83	dom	0.37(0.03–4.24)	0.43	0.16(0.01–2.65)	0.20
*CSK*	15	rs1378942	intron	C	0.52	rec	1.84(0.80–4.23)	0.15	2.06(0.66–6.43)	0.22
*BAG6*	6	rs805303	intron	G	0.58	dom	0.75(0.31–1.85)	0.53	0.53(0.17–1.65)	0.27
*CACNB2*	10	rs11014171^	intron	C	0.75	rec	1.19(0.68–3.26)	0.32	1.51(0.58–3.91)	0.40
*MTHFR*	1	rs17367504	intron	A	0.90	add	1.69(0.65–4.37)	0.28	1.46(0.46–4.65)	0.52
*CACNB2*	10	rs4373814	5′ flanking	C	0.47	dom	1.37(0.57–3.25)	0.48	1.34(0.49–3.64)	0.57
*HFE*	6	rs1799945	intronic/missense	G	0.084	dom	0.64(0.22–1.86)	0.41	0.84(0.22–3.15)	0.80
*NPR3*	5	rs1173771	3′ flanking	G	0.64	rec	1.09(0.50–2.39)	0.83	1.06(0.40–2.83)	0.91

*Adjusted for follow-up time, age at diagnosis, gender, race, hypertension, anthracycline dose, chest radiation, cancer site.

^Serving as a proxy for rs1813353 (r^2^ = 1).

Abbreviations: AF- allele frequency in study population, model – model of inheritance, add – additive, dom – dominant, rec – recessive.

### Gene Expression in iPSC-Cardiomyocytes

**Figure 1:**
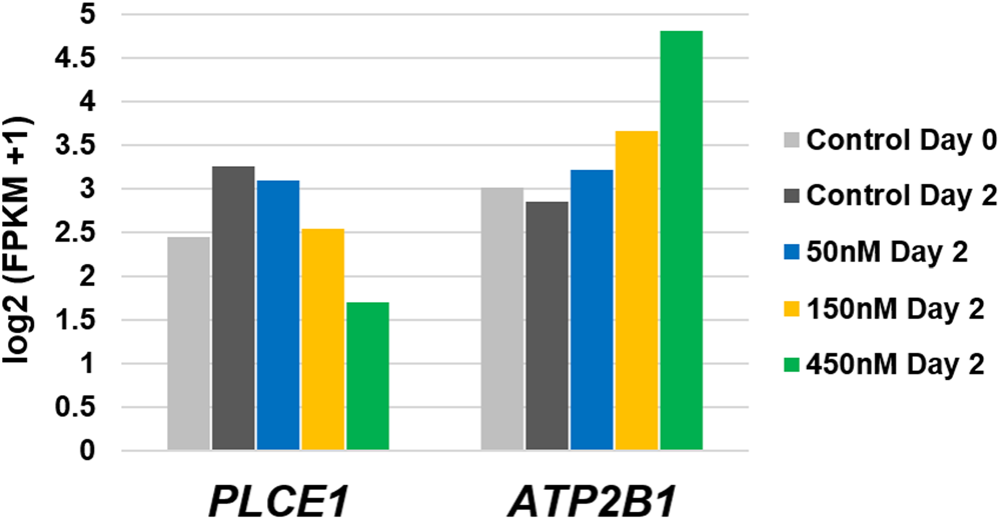
Gene Expression Levels of *PLCE1* and *ATP2B1* in iPSC-Cardiomyocytes Exposed to Doxorubicin. iPSC-cardiomyocytes were cultured for two days to establish contractility (“Day 0”), followed by a 2-day exposure to various doses of doxorubicin. Gene expression for both genes was measured by RNAseq and expressed as log_2_(FKPM + 1).

Analysis of RNAseq data generated from iPSC-cardiomyocytes exposed to increasing doses of doxorubicin identified that expression of both *PLCE1* and *ATP2B1* was anthracycline-dependent (Fig. 1). *PLCE1* gene expression levels decreased by dose after two days of exposure to doxorubicin at 50 nM, 150 nM, and 450 nM compared to untreated cells. Under the same conditions, *ATP2B1* gene expression levels were elevated with increasing doses of doxorubicin. These doses have previously been shown to have an effect on iPSC-cardiomyocyte contractility, yet not alter cell viability^15^.

### Functional Prediction

In a lymphoblastoid cell line model system, Gamazon *et al*. previously investigated relationships between genetic variants, gene expression, and cytotoxicity to daunorubicin^16^. Querying this dataset, a significant correlation between *PLCE1*:rs932764 and daunorubicin response (expressed as IC_50_) was observed (P = 0.0080), as well as four other proxy variants in *PLCE1* (rs10786152, rs2901761, rs731141, and rs9663362). rs10786152 was also predicted by SNiPA to be located in putative regulatory region defined by open chromatin and HaploReg predicted that this same variant was associated with enhancer histone marks in skeletal muscle myoblasts.

The Gamazon *et al*. dataset also demonstrated a significant correlation between *ATP2B1* gene expression and daunorubicin IC_50_ values in lymphoblastoid cell lines (P = 0.0079)^16^. Significant cis-eQTL relationships^17^ in whole blood for two other genes located near *ATP2B1* on chr12q21 – *GALNT4* and *POC1B –* were identified for the significant directly genotyped variant in *ATP2B1*, rs17249754, and nine other proxy variants. This cluster of variants in high LD is located within putative regulatory regions defined by open chromatin, promoter regulatory clusters, DNase hyper-sensitivity sites, and enhancer histone marks.

## Discussion

It is estimated that there will be approximately 500,000 childhood cancer survivors by 2020^1^, resulting in a parallel increase in the incidence of late-effects in this population. As many as 65% of individuals who were treated with anthracycline-based chemotherapy regimens will have echocardiographic evidence of impaired contraction^18^. This increased risk of cardiotoxicity underscores the importance of increased understanding of the predictors of late-effects and the underlying mechanisms. In this study, two established hypertension-susceptibility alleles identified in the general population (*PLCE1*:rs932764 and *ATP2B1*:rs17249754) were also identified as predictors of cardiotoxicity risk in long-term childhood cancer survivors exposed to anthracyclines. However, we uncovered an unexpected inverse relationship between these two endpoints with the alleles that increased risk of hypertension conferring a protective effect on anthracycline-related cardiotoxicity. Gene expression analyses in iPSC-derived cardiomyocytes and *in silico* analysis support the role of both *PLCE1* and *ATP2B1* in anthracycline response.

Our analysis identified that the hypertension-susceptibility G allele of rs932764 located in an intron of *PLCE1*, which encodes for phospholipase C epsilon (PLCε), is associated with a significant reduction in risk of cardiotoxicity in our survivor population. Phospholipase C is a family of proteins responsible for second messenger signaling with a wide range of downstream functions within the cell, including induction of signaling through the Ca2+ and protein kinase C pathways^19–21^. In the heart, the role of PLCε is emerging. In a screen for genes involved in heart failure, Wang *et al*. found that *PLCE1* expression was upregulated in tissues obtained from failing human hearts, while PLCε knockout mice showed increased susceptibility to hypertrophy, decreased contractility following stimulation, and decreased sensitivity to beta-adrenergic receptor mediated Ca2+ signaling^22^. Xiang *et al*. also demonstrated a cardioprotective role for PLCε from oxidative stress through activation of protein kinase D and subsequent downstream maintenance of mitochondrial integrity^23^. Intriguingly, one mechanism by which anthracyclines damage the heart is through increases in reactive oxygen species^24^. In this scenario, upregulation of *PLCE1* in the heart would serve as a protective mechanism against oxidative stress. Other studies suggest a more complex function of PLCε in the heart. In an *in vivo* mouse model of hypertrophy, the conditional loss of PLCε expression was protective from TAC banding-induced hypertrophy^25, 26^. Further, overexpression of PLCε resulted in cellular hypertrophy, likely secondary to increased lipase catalytic function^25^. Together, the evidence suggest that PLCε action in the heart is complex and likely serves as a molecular integrator of numerous exogenous signals^27^. To begin to elucidate the role of PLCε in the development of anthracycline-induced cardiotoxicity, we showed that *PLCE1* displayed an inverse dose-dependent expression changes in response to anthracycline exposure in human iPSC-cardiomyocytes. Our findings support a protective role for PLCε in anthracycline-dependent cardiotoxicity. Further analyses to determine genotype-phenotype relationships with PLCε and rs932764 in the heart and how this relationship is potentially mediated by hypertension are warranted.

In addition to *PLCE1*, carriers of the hypertension risk allele in *ATP2B1* were at a reduced risk of cardiotoxicity. *ATP2B1* encodes for plasma membrane calcium ATPase isoform 1 (PMCA1) and is a Ca2+ ATPase that functions as a high-affinity, low-capacity Ca2+ plasma membrane pump that is known to be expressed in the heart^28, 29^. It is responsible for maintaining low levels of Ca2+ in the cell during “resting” conditions^30, 31^. Altered Ca2+ homeostasis, particularly increased cytosolic Ca2+ and stored-Ca2+ leak, is a known trigger for pathologic cardiac remodeling that can underlie cardiac hypertrophy and impaired systolic heart failure^32–34^. Indeed, protein expression levels of PMCA1 are decreased in heart tissue from individuals with heart failure compared to healthy hearts^35^. Similarly to the findings for *PLCE1*, we observed *ATP2B1* expression in the iPSC-cardiomyocyte model system to be anthracycline-dependent with increases in gene expression in a dose-dependent manner and *in silico* prediction identified a significant correlation between *ATP2B1* expression and daunorubicin cytotoxicity. rs17249754 is located in the 5′-flanking region of ATP2B1 and is in strong LD with a cluster of variants that are located in putative regulatory elements. A previous study demonstrated that one of these variants, rs11105378 (r^2^ = 0.91 with rs17249754), was associated with *ATP2B1* expression in umbilical artery smooth muscle cells^36^. Our results provide further support of the importance of this gene in cardiovascular function and extend to demonstrate the impact of alterations within this gene on the development of cardiotoxicity. As with *PLCE1*:rs932764, genotype-phenotype correlation analyses are warranted to better understand how rs17249754 alters *ATP2B1* function in the heart.

This inverse relationship between *PLCE1*:rs932764 and *ATP2B1*:rs17249754 associations with hypertension and cardiotoxicity suggests that the biological function of these two genes are different in the setting of hypertension verses response to anthracyclines resulting in cardiotoxicity. Indeed, the pathophysiology of these two disease states are different with different cell types involved. Hypertension is a result of changes in the endovascular/smooth muscle with renal involvement, while cardiotoxicity primarily involves the cardiomyocyte. In support of this dual role, previous studies have shown that intracellular Ca2+ regulation by ATP2B1 has a role in vascular tone and blood pressure^37^. Mice homozygous for loss of *ATP2B1* are embryonic lethal^38^ and recent knockdown experiments in mice have shown that *ATP2B1* expression is needed for regulation of blood pressure^39–41^. Furthermore, mutations in *PLCE1* have been associated with nephrotic syndrome type 3 ^42^. Alternatively, there is potential that the genotyped polymorphisms serve as surrogate markers for distinct causal variants that have cell-type specific effects. In this scenario, the causal variants tagged by *PLCE1*:rs932764 and *ATP2B1*:rs17249754 in the cardiomyocyte would disrupt cell-type specific regulatory factors that respond to anthracycline exposure, while in the endovascular cell this regulatory element would not be in play due to an absence of these heart-specific factors. A deeper investigation of the mechanisms underlying the associations with cardiotoxicity and hypertension are needed to provide clarity regarding the roles of these loci and genes in both disease states.

A strength of this study was the detailed longitudinal echocardiographic data available to clearly define the case and control populations. Furthermore, all study participants were followed-up at a single institution, reducing the introduction of variability due to differences in follow-up protocols and reading of the echocardiograms. The designation of case and control were such that we included only those with clear, clinically defined cardiotoxicity, increasing our likelihood of identifying a significant association based on sampling at the extremes of the phenotype. This strategy to define cases and controls based on sequential echocardiograms also reduces the possibility of controls having sub-clinical, asymptomatic cardiotoxicity that has not yet been diagnosed. As our control population was followed up a slightly shorter duration compared to cases, having echocardiographic confirmation of function helps to reduce the number of undiagnosed study participants that could potentially meet the criteria for cases with additional follow-up. Cases and controls were matched on mean anthracycline dose to minimize the effect of this major risk factor driving the development of cardiotoxicity. However, even with matching resulting in this variable not being statistically significantly different between the two groups, the cases did have slightly higher anthracycline exposure. This may have a slight impact on our results, although both cases and controls were classified as “high risk” based on heart failure risk scores^44^. All patients who received anthracycline-based chemotherapy were also treated with other agents as indicated by cancer diagnosis. Although no patients were treated with targeted agents with known cardiotoxicity or with common chemotherapy agents that have been associated with cardiotoxicity in pediatric epidemiological studies except at very high doses, there is a possibility that our associations may be modified by these other agents. Larger sample sizes will be required to clarify any potential drug-drug-gene interactions. Our genetic association findings have high biological plausibility and are further supported by our RNAseq data from iPSC-derived cardiomyocytes exposed to anthracycline and *in silico* functional prediction analyses, providing functional validation of the findings. Further studies in other, larger populations of long-term childhood cancer survivors exposed to anthracyclines are warranted to provide validation of the genetic associations. These larger studies would also enable deeper investigation into the interactions between these genetic markers with anthracycline dose and hypertension.

In conclusion, two genetic variants in *PLCE1* and *ATP2B1* are inversely associated with hypertension and cardiotoxicity susceptibility. Gene expression demonstrated a link with anthracycline drug response phenotypes in human iPSC-cardiomyocytes and *in sillico* analysis supported the same in a lymphoblastoid cell line model system. With current cardiotoxicity risk prediction approaches insufficient to accurately assess risk in long-term childhood cancer survivor populations, the current study provides additional potential candidate loci for risk, while also elucidating potential mechanisms underlying the development of cardiotoxicity.

## Methods

### Study Population

Childhood cancer survivors were recruited from MD Anderson Cancer Center between 2004 and 2007. All participants were more than 5 years post-diagnosis, off therapy, and had received anthracyclines as part of their cancer treatment. Patients who had received an allogeneic stem cell transplant were excluded from the analysis. Each was followed in the Childhood Cancer Survivor (CCS) Clinic at MD Anderson with echocardiographic screening conducted regularly every 1 to 5 years as recommended by the risk-based COG survivorship guidelines. Patient demographics, cancer diagnosis, age at diagnosis, treatment regimens including total anthracyclines dose, diagnosis of hypertension, echocardiographic measurements, and dates of all echocardiograms were collected. Anthracycline doses were standardized to doxorubicin equivalents using the COG guidelines to calculate a total anthracycline dose for patients who may have received more than one type of anthracycline^43^. Heart failure risk scores were calculated for each participant^44^. DNA was extracted from blood specimens collected and banked according to standard procedures. The study was approved by the Institutional Review Board of MD Anderson and written informed consent obtained from all study participants. All methods and analyses were carried out in accordance with this approval.

Based on echocardiograms and documentation in the chart by cardiologists at MD Anderson and the CCS Clinic, patients who received anthracyclines were classified as “cases” and “controls” (Table 1) based on the following criteria: 1) Cases either had EF 45–50% and had symptoms and other echocardiogram findings considered by a cardiologist to warrant cardiac medications; or had EF ≤ 45% and/or shortening fraction (SF) ≤ 25% on at least two echocardiograms. All patients included because they were started on cardiac medications had chart review to assure that medications were started for cardiac dysfunction and not hypertension without echocardiogram abnormalities. Patients who had a single low EF or SF that returned to EF > 55% without the use of cardiac medications were not included as cases; 2) Controls - EF > 55% and SF > 28%, with at least two echocardiograms obtained more than 5 years off treatment. A patient with a single discrepant low EF or SF with subsequent normal echocardiograms not on medications were considered a control. Only medications that were indicated in the medical record for treatment of impaired cardiac function and not for treatment of hypertension were coded as cardiac medications. Cases and controls were frequency matched by age at diagnosis, anthracycline dose, gender, chest radiation, and cancer type.

### Genetic Variant Selection and Genotyping

Twelve index genetic variants were selected for genotyping based on previous evidence of significant association with hypertension from a GWAS involving >200,000 individuals in the general population^14^. TaqMan Genotyping Assays (ABI, Foster City, CA) were available for 11 of the 12 loci, with a variant (rs11014171) in high linkage disequilibrium to rs1813353 (r^2^ = 1) selected for genotyping as a proxy for that locus. Genotyping was performed according to standard protocols on the ABI 7900HT platform. Quality control measures were in place, including negative controls and replicates. All assays were performed blinded to the cardiotoxicity status of the individuals.

### Statistical Analysis

Student’s t-tests or chi-squared tests were used to compare characteristics of case and control populations. Odds ratios (ORs) and 95% Confidence Intervals (CIs) for each polymorphism were calculated using univariate and multivariate unconditional logistic regression with the hypertension risk allele^14^ coded as the potential risk allele for the analysis. All multivariate analyses included adjustment for follow-up time, age at diagnosis, gender, race, hypertension, anthracycline dose, chest radiation, and cancer site. Since we had no prior knowledge regarding the underlying model of inheritance predisposing to cardiotoxicity risk, we assessed the effect of each variant under the dominant, recessive, and additive models. The model with the lowest p-value was considered the primary model for that locus with a P value of 0.05 set as the threshold of significance.

### iPCS-Cardiomyocyte Culturing and Exposure to Doxorubicin

Cardiomyocytes generated from a healthy individual were obtained from Cellular Dynamics (Madison, WI). Frozen cells were thawed, plated, and cultured following the iCell Cardiomyocytes^2^ protocol. Briefly, approximately 24 hours prior to thawing and plating of cells into 12-well plates, each well was coated with fibronectin solution (1 mg/mL) and incubated overnight at 37 °C. Cells were thawed, diluted in Plating Media, and counted by trypan blue exclusion cell viability assay (Gibco). Re-suspended cells were seeded at a density of 6 × 10^5^ cells/well immediately following aspiration of fibronectin solution from each well, then incubated for 4 hours at 37 °C. Cells were then cultured in Maintenance Media for the remainder of the experiment. Following 48 hours in culture, cells were treated with doxorubicin (50, 150, and 450 nM) or left untreated as a control. On day 2 (post-treatment), cells were harvested for RNA isolation and flash frozen for storage at −80 °C. RNA was isolated from each cell pellet using the RNeasy kit (Qiagen). RNA concentrations were assessed using the Nanodrop Spectrophotometer.

### RNAseq of iPSC-Cardiomyocytes

Each RNA specimen was diluted in 100 uL RNase-free water to a final concentration of 15 ng/µL. RNAseq was performed in MD Anderson’s Sequencing and Microarray Facility using the Illumina TruSeq Stranded Total RNA Library Prep Kit with Ribo-Zero Gold. The 13 strand-specific libraries were pooled and divided across two lanes for sequencing with the Illumina HiSeq. 3000. Demultiplexed reads of ~100 bp length were generated for each sample. The FASTQ files generated per lane were combined for each sample and aligned to GRCh37 using STAR^45^. The resulting BAM files were evaluated by metrics generated from RNA-SeQC for quality control^46^. Transcripts were assembled with CUFFLINKS and CUFFMERGE from the aligned reads. CUFFNORM was used to generate normalized expression profiles^47^. Read counts were normalized using fragments per kilobase of transcript per million mapped fragments (FPKM) and gene expression analyzed as log_2_(FPKM + 1).

### *In silico* Functional Genomic Analysis

The Proxy Search function of SNAP^48^ was used to identify additional variants in high LD (r^2^ > 0.8) with the directly genotyped 12 variants. A total of 95 variants were identified and inputted into HaploReg v2^49^ and SNiPA^50^ for functional predictions. The PACdb database was queried to identify potential correlations between the variant or host gene identified in the association analysis and anthracycline-induced cytotoxicity in lymphoblastoid cell lines^16^.

## References

[1] Robison LL, Hudson MM (2014). Survivors of childhood and adolescent cancer: life-long risks and responsibilities. Nat Rev Cancer.

[2] Lotrionte M (2013). Review and meta-analysis of incidence and clinical predictors of anthracycline cardiotoxicity. Am J Cardiol.

[3] Lipshultz SE (2013). Long-term cardiovascular toxicity in children, adolescents, and young adults who receive cancer therapy: pathophysiology, course, monitoring, management, prevention, and research directions: a scientific statement from the American Heart Association. Circulation.

[4] Leger K (2015). Subclinical cardiotoxicity in childhood cancer survivors exposed to very low dose anthracycline therapy. Pediatr Blood Cancer.

[5] Duan S (2007). Mapping genes that contribute to daunorubicin-induced cytotoxicity. Cancer Res.

[6] Wang X (2016). CELF4 Variant and Anthracycline-Related Cardiomyopathy: A Children’s Oncology Group Genome-Wide Association Study. J Clin Oncol.

[7] Aminkeng F (2015). A coding variant in RARG confers susceptibility to anthracycline-induced cardiotoxicity in childhood cancer. Nat Genet.

[8] Visscher H (2012). Pharmacogenomic prediction of anthracycline-induced cardiotoxicity in children. J Clin Oncol.

[9] Visscher H (2013). Validation of variants in SLC28A3 and UGT1A6 as genetic markers predictive of anthracycline-induced cardiotoxicity in children. Pediatr Blood Cancer.

[10] Blanco JG (2008). Genetic polymorphisms in the carbonyl reductase 3 gene CBR3 and the NAD(P)H:quinone oxidoreductase 1 gene NQO1 in patients who developed anthracycline-related congestive heart failure after childhood cancer. Cancer.

[11] Blanco JG (2012). Anthracycline-related cardiomyopathy after childhood cancer: role of polymorphisms in carbonyl reductase genes–a report from the Children’s Oncology Group. J Clin Oncol.

[12] Wang X (2014). Hyaluronan Synthase 3 Variant and Anthracycline-Related Cardiomyopathy: A Report From the Children’s Oncology Group. J Clin Oncol.

[13] Armstrong GT (2013). Modifiable risk factors and major cardiac events among adult survivors of childhood cancer. J Clin Oncol.

[14] Ehret GB (2011). Genetic variants in novel pathways influence blood pressure and cardiovascular disease risk. Nature.

[15] Sirenko O (2013). Assessment of beating parameters in human induced pluripotent stem cells enables quantitative *in vitro* screening for cardiotoxicity. Toxicol Appl Pharmacol.

[16] Gamazon ER (2010). PACdb: a database for cell-based pharmacogenomics. Pharmacogenet Genomics.

[17] Westra HJ (2015). Cell Specific eQTL Analysis without Sorting Cells. PLoS Genet.

[18] Grenier MA, Lipshultz SE (1998). Epidemiology of anthracycline cardiotoxicity in children and adults. Semin Oncol.

[19] Bunney TD, Katan M (2006). Phospholipase C epsilon: linking second messengers and small GTPases. Trends Cell Biol.

[20] Fukami K (2002). Structure, regulation, and function of phospholipase C isozymes. J Biochem.

[21] Rebecchi MJ, Pentyala SN (2000). Structure, function, and control of phosphoinositide-specific phospholipase C. Physiol Rev.

[22] Wang H (2005). Phospholipase C epsilon modulates beta-adrenergic receptor-dependent cardiac contraction and inhibits cardiac hypertrophy. Circ Res.

[23] Xiang SY (2013). PLCepsilon, PKD1, and SSH1L transduce RhoA signaling to protect mitochondria from oxidative stress in the heart. Sci Signal.

[24] Sawyer DB, Peng X, Chen B, Pentassuglia L, Lim CC (2010). Mechanisms of anthracycline cardiac injury: can we identify strategies for cardioprotection?. Prog Cardiovasc Dis.

[25] Zhang L, Malik S, Kelley GG, Kapiloff MS, Smrcka AV (2011). Phospholipase C epsilon scaffolds to muscle-specific A kinase anchoring protein (mAKAPbeta) and integrates multiple hypertrophic stimuli in cardiac myocytes. J Biol Chem.

[26] Zhang L (2013). Phospholipase Cepsilon hydrolyzes perinuclear phosphatidylinositol 4-phosphate to regulate cardiac hypertrophy. Cell.

[27] Smrcka AV, Brown JH, Holz GG (2012). Role of phospholipase Cepsilon in physiological phosphoinositide signaling networks. Cell Signal.

[28] Brandt P, Neve RL, Kammesheidt A, Rhoads RE, Vanaman TC (1992). Analysis of the tissue-specific distribution of mRNAs encoding the plasma membrane calcium-pumping ATPases and characterization of an alternately spliced form of PMCA4 at the cDNA and genomic levels. J Biol Chem.

[29] Santiago-Garcia J, Mas-Oliva J, Saavedra D, Zarain-Herzberg A (1996). Analysis of mRNA expression and cloning of a novel plasma membrane Ca(2 + )-ATPase splice variant in human heart. Mol Cell Biochem.

[30] Brini, M. & Carafoli, E. The plasma membrane Ca(2) + ATPase and the plasma membrane sodium calcium exchanger cooperate in the regulation of cell calcium. *Cold Spring Harb Perspect Biol* **3**, doi:10.1101/cshperspect.a004168 (2011). 10.1101/cshperspect.a004168 PMC3039526 21421919

[31] Bers DM (2002). Cardiac excitation-contraction coupling. Nature.

[32] Bers DM (2014). Cardiac sarcoplasmic reticulum calcium leak: basis and roles in cardiac dysfunction. Annu Rev Physiol.

[33] Landstrom AP (2011). Junctophilin-2 expression silencing causes cardiocyte hypertrophy and abnormal intracellular calcium-handling. Circ Heart Fail.

[34] van Oort RJ (2011). Disrupted junctional membrane complexes and hyperactive ryanodine receptors after acute junctophilin knockdown in mice. Circulation.

[35] Borlak J, Thum T (2003). Hallmarks of ion channel gene expression in end-stage heart failure. FASEB J.

[36] Tabara Y (2010). Common variants in the ATP2B1 gene are associated with susceptibility to hypertension: the Japanese Millennium Genome Project. Hypertension.

[37] Gros R (2003). Plasma membrane calcium ATPase overexpression in arterial smooth muscle increases vasomotor responsiveness and blood pressure. Circ Res.

[38] Okunade GW (2004). Targeted ablation of plasma membrane Ca2 + -ATPase (PMCA) 1 and 4 indicates a major housekeeping function for PMCA1 and a critical role in hyperactivated sperm motility and male fertility for PMCA4. J Biol Chem.

[39] Kobayashi Y (2012). Mice lacking hypertension candidate gene ATP2B1 in vascular smooth muscle cells show significant blood pressure elevation. Hypertension.

[40] Fujiwara, A. *et al*. Impaired nitric oxide production and increased blood pressure in systemic heterozygous ATP2B1 null mice. *J Hypertens* **32**, 1415-1423; discussion 1423, doi:10.1097/HJH.0000000000000206 (2014). 10.1097/HJH.0000000000000206 24805951

[41] Shin YB (2013). Silencing of Atp2b1 increases blood pressure through vasoconstriction. J Hypertens.

[42] Hinkes B (2006). Positional cloning uncovers mutations in PLCE1 responsible for a nephrotic syndrome variant that may be reversible. Nat Genet.

[43] Shankar SM (2008). Monitoring for cardiovascular disease in survivors of childhood cancer: report from the Cardiovascular Disease Task Force of the Children’s Oncology Group. Pediatrics.

[44] Chow EJ (2015). Individual prediction of heart failure among childhood cancer survivors. J Clin Oncol.

[45] Dobin A, Gingeras TR (2015). Mapping RNA-seq Reads with STAR. Curr Protoc Bioinformatics.

[46] Wang L, Wang S, Li W (2012). RSeQC: quality control of RNA-seq experiments. Bioinformatics.

[47] Trapnell C (2012). Differential gene and transcript expression analysis of RNA-seq experiments with TopHat and Cufflinks. Nat Protoc.

[48] Johnson AD (2008). SNAP: a web-based tool for identification and annotation of proxy SNPs using HapMap. Bioinformatics.

[49] Ward LD, Kellis M (2012). HaploReg: a resource for exploring chromatin states, conservation, and regulatory motif alterations within sets of genetically linked variants. Nucleic Acids Res.

[50] Arnold M, Raffler J, Pfeufer A, Suhre K, Kastenmuller G (2015). SNiPA: an interactive, genetic variant-centered annotation browser. Bioinformatics.

